# Immune Check-Point Inhibitors and Standard Chemoradiotherapy in Definitive Head and Neck Cancer Treatment

**DOI:** 10.3390/jpm11050393

**Published:** 2021-05-10

**Authors:** Francesca De Felice, Daniela Musio, Vincenzo Tombolini

**Affiliations:** Department of Radiotherapy, Policlinico Umberto I, “Sapienza” University of Rome, 00161 Rome, Italy; daniela.musio@uniroma1.it (D.M.); vincenzo.tombolini@uniroma1.it (V.T.)

**Keywords:** immunotherapy, check-point inhibitors, head neck cancer, chemoradiotherapy, HPV, definitive treatment, outcomes

## Abstract

In head and neck cancer management, there is a need for tailored approaches to optimally implement clinical outcomes. Based on the assumption that efficacy and long-term toxicity are not satisfactory for standard concurrent platinum-based chemoradiotherapy, several trials have been designed to test whether induction immunotherapy and/or concomitant immunotherapy and radiotherapy result in improved survival and toxicity outcomes. Here, we present an overview of the most recent concomitant therapeutic strategies for head and neck cancer, focusing on the knowledge available regarding check-point inhibitors. The aim is to present the characteristics of the main check-point inhibitors and to summarize the clinical trials on the combination of immune check-point inhibitors and (chemo)radiotherapy in the definitive HNC setting, in order to provide a useful clinical tool for further research.

## 1. Introduction

Given the predicted rising incidence of head and neck cancer (HNC) up to 856,000 cases by 2035, challenges to implementing and improving treatment approaches have to be faced in future decade [1]. At present, the surgical approach, radiotherapy, systemic therapy, or a combination of these modalities are the standard of care in HNC management [2]. The optimal treatment is chosen on tha basis of primary location and stage disease at diagnosis, and prognosis remains poor especially for locally advanced disease and for recurrent/metastatic cases [2,3]. In the last years, immunotherapy, primarily check-point inhibitors, has become an established pillar for the treatment of several human malignancies [4,5,6]. Nivolumab and pembrolizumab are both anti-programmed death-1 (PD-1) antibodies. Firstly approved by the Food and Drug Administration (FDA) for patients with metastatic melanoma in 2011 [7], currently these molecules represent first-line (pembrolizumab) or subsequent-line (nivolumab) palliative HNC treatment options [2,8,9]. In this context, we aim to emphasize the current developments in the research on immune check-point inhibitors in a curative HNC setting. We also describe and compare check-point inhibitors mechanisms of action, as well as their main pharmacokinetics properties and their immune-related toxicities.

## 2. Literature Search

**Figure 1:**
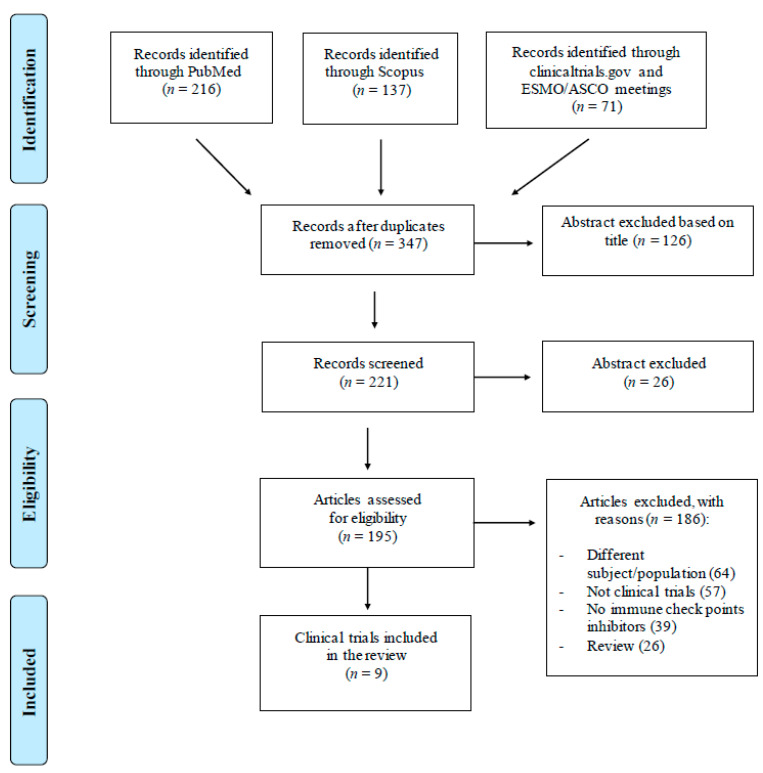
PRISMA flow diagram describing the data collection process following the PRISMA convention.

Literature search was performed in accordance with the Preferred Reporting Items for Systematic Reviews and Meta-Analyses (PRISMA) guidelines. PubMed and Scopus databases were searched, using the following keywords: “head and neck cancer”, “immunotherapy”, “immune check-point”, “inhibitors”, “nivolumab”, “pembrolizumab”, “ipilimumab”, “avelumab”, “durvalumab”, “PD-1”, “PD-L1”, “CTLA-4”. Inclusion criteria included immune checkpoint inhibition in the setting of radical head and neck squamous cell carcinoma treatment. Only papers written in English were considered. Additional relevant publications were manually searched using the proceedings of the annual meeting of the European Society for Medical Oncology (ESMO) and the American Society of Clinical Oncology (ASCO) and clinicaltrials.gov. Search was performed up to October 2020. Regarding the section of clinical trials using check-point inhibitors in curative HNC, the literature search process yielded 216 results on PubMed, 137 results on Scopus, and 71 results on clinicaltrials.gov. Screening the abstracts of the annual meetings of ESMO and ASCO added 11 results. Sixty-seven duplicates were removed. In total, 221 results were screened for eligibility. Most papers (*n* = 186) did not fulfill the inclusion criteria mainly due to (i) different subjects/populations (*n* = 64), (ii) not a clinical trial (*n* = 57), (iii) no immune check-point inhibitors (*n* = 39). Finally, 9 clinical trials testing immune check-point inhibitors in addition to definitive chemoradiotherapy in head and neck cancer treatment were included (Figure 1).

## 3. Immune Check-Point Inhibitors

Cancer cells have numerous mechanisms to evade local immune attack, including the upregulation of immune check-point protein expression. The end result is cancer progression that clinically manifests as a tumor [10]. At present, immune check-point inhibitors represent the most successful immunotherapeutic approach due to their peculiar ability to target lymphocyte receptors, as opposed to the current targeting therapy, such as that based on bevacizumab, trastuzumab, and cetuximab, that acts directly on tumor cells. In fact, immune check-point proteins are physiologically expressed by activated lymphocytes and are triggered by ligand–receptor interactions. Considering that the immune system response is regulated by a balance between stimulatory and inhibitory signals, each immune check-point can be properly blocked by agonist (stimulatory signals) or antagonist (inhibitory signals) antibodies or modulated by recombinant forms of ligands or receptors [5,11].

We summarize actual check-point inhibitors, their target, mode of action, toxicities, and clinical evidence in HNC in Table 1, in order a reader-friendly overview. To note, ipilimumab, durvalumab, avelumab, atezolizumab, and tremelilumab are not used for HNC at present, but we included them given that they are raising attention, as reported in the clinical evidence section.

### 3.1. Nivolumab

Nivolumab is a fully human IgG4 monoclonal antibody that targets PD-1. It binds to the PD-1 receptor, releasing PD-1 pathway-mediated inhibition of the immune response, including the anti-tumor immune response [12]. At present, it is recommended for patients with recurrent or metastatic squamous cell carcinoma of the head and neck with ongoing disease progression or after a platinum-based therapy [2,12]. Nivolumab is administered by a 30 min intravenous infusion at 240 mg every 2 weeks or 480 mg every 4 weeks until tumor progression or intolerable toxicity [12]. Steady-state concentration is reached by 12 weeks; the mean elimination half-life (t_1/2_) is 25 days, and the clearance rate is 8.2 mL/h. Nivolumab clearance is directly related to increasing body weight, whereas it is not influenced by other patient (age, gender, race) and tumor (type, size, PD-L1 expression) characteristics [12].

### 3.2. Pembrolizumab

Pembrolizumab is a humanized IgG4 monoclonal antibody against PD-1 receptor on T cells. It derives from a nonhuman species protein whose sequences have been modified to increase their similarity to human antibody variants [10]. Its mechanism of action is similar to that of nivolumab [13]. It is indicated (i) in combination with platinum and fluorouracil for the first-line treatment of patients with metastatic or with unresectable, recurrent squamous cell carcinoma of the head and neck; (ii) as a single agent for the first-line treatment of patients with metastatic or unresectable, recurrent squamous cell carcinoma of the head and neck whose tumors express PD-L1—combined positive score (CPS) ≥1—as determined by an FDA-approved test; (iii) as a single agent for the treatment of patients with recurrent or metastatic squamous cell carcinoma of the head and neck with ongoing disease progression or after platinum-containing chemotherapy. It should be clarified that, originally, first-line pembrolizumab in combination with chemotherapy was not universally indicated. For instance, in the United Kingdom, the drug was initially rejected in January 2020 due to uncertainties in the clinical trial data but has now been approved by the National Institute of Health and Care Excellence (NICE) based on additional data (https://www.cancerresearchuk.org, accessed on 15 March 2021).

Pembrolizumab is administered as an intravenous infusion over 30 min at 200 mg every 3 weeks or 400 mg every 6 weeks until disease progression or unacceptable toxicity [13]. Steady-state concentrations are reached by 16 weeks; the mean elimination half-life (t_1/2_) is 22 days, and the clearance rate is 9.2 mL/h. Its clearance only increases with increasing body weight. Pembrolizumab has not been studied in patients with moderate or severe hepatic impairment [13].

### 3.3. Durvalumab

Durvalumab is a human immunoglobulin G1 kappa (IgG1κ) monoclonal antibody that blocks the interaction of programmed cell death ligand 1 (PD-L1) with the PD-1 and CD80 molecules [14], without inducing antibody-dependent cell-mediated cytotoxicity. Steady-state concentration is achieved at 16 weeks, and its half-life is approximately 17 days. Durvalumab is administered in infusion solution intravenously 10 mg/kg over 60 min every 2 weeks. It was approved in 2017 for the treatment of patients with locally advanced or metastatic urothelial carcinoma [15].

### 3.4. Ipilimumab

Ipilimumab is a fully human monoclonal IgG1 antibody that antagonizes cytotoxic T lymphocyte-associated antigen 4 (CTLA-4), increasing the activation and proliferation of T cells [16]. It is the parent immune check-point inhibitor, firstly approved by FDA in 2011 to treat unresectable or metastatic melanoma patients [14]. Typically, ipilimumab is administered at 3 mg/kg infused over 90 min every 3 weeks for a total of four doses in a metastatic setting [16]. Steady-state concentration is reached by the third dose; t_1/2_ is 15.4 days, and the clearance rate is 16.8 mL/h [16]. Only body weight has a clinical important effect on its clearance.

### 3.5. Avelumab

Avelumab is a fully human anti-PD-L1 IgG1 monoclonal antibody, with dual engagement of the adaptive and innate immune systems. It releases the suppression of the T cell-mediated antitumor immune response by blocking the interaction of PD-L1 with PD-1 receptors (adaptive immune response) and induces NK cell-mediated direct tumor cell lysis via antibody-dependent cell-mediated cytotoxicity (innate immune response) [17]. The recommended dose and schedule of avelumab is 10 mg/kg as an intravenous infusion over 60 min every 2 weeks until disease progression or unacceptable toxicity [18]. Its pharmacokinetics has been studied in 1629 patients who received doses ranging from 1 to 20 mg/kg every 2 weeks. Steady-state concentrations were reached after 4–6 weeks. The primary elimination mechanism is proteolytic degradation. Total systemic clearance was 0.59 L/day, and terminal half-life (t_1/2_) was 6.1 days in patients receiving 10 mg/kg.

### 3.6. Atezolizumab

Atezolizumab is a monoclonal antibody that binds to PD-L1 and blocks the PD-1/PD-L1 pathway [19]. Clearance was 0.20 L/day, volume of distribution at steady state was 6.9 L, and terminal half-life (t_1/2_) was 27 days. Its infusion should be delivered intravenously over 60 min. Steady state was achieved after 6–9 weeks following multiple doses, and its clearance was found to decrease over time. Atezolizumab is indicated for the treatment of adult patients with locally advanced/metastatic urothelial carcinoma, patients with metastatic non-small cell lung cancer or with extensive-stage small cell lung cancer, patients with unresectable locally advanced or metastatic triple-negative breast cancer, patients with unresectable or metastatic hepatocellular carcinoma, and patients with metastatic melanoma [19].

### 3.7. Tremelimumab

Tremelimumab is an investigational, fully human monoclonal antibody that binds to the CTLA-4 molecule [18]. Tremelimumab is administered at a dose of 15 mg/kg as an intravenous infusion every 90 days until tumor progression or severe toxicity. It exhibited a terminal phase half-life (t_1/2_) of 22.1 days [20].

## 4. Safety and Tolerability

Usually, immune check-point inhibitors have an acceptable toxicity profile. Their adverse events (AEs) are primarily consistent with their mechanism of action. CTLA-4 AEs are mainly dose-related whereas PD-1 toxicity is not dose-associated. Consequently, AEs of CTLA-4 blockade appear to be more severe than those of PD-1 blockade, accounting for a severe grade frequency of 10–20% versus 1–2%. Both general and organ-specific AEs are reported. The main general AEs are fatigue and injection-site reactions. Organ-specific AEs may include dermatologic toxicity(erythematous rash on the extremities and trunk), gastro-intestinal toxicity(mucositis, diarrhea and proctocolitis)hepatic and endocrine toxicity(endocrinopathies affecting the pituitary gland, adrenal gland and thyroid gland). A direct correlation between AEs occurrence/severity and tumor response has been described [21].

For clarity, the following AEs description came from each immune check-point inhibitor prescribing information.

### 4.1. Nivolumab

Most frequent AEs (≥20% of cases) are fatigue, rash, musculoskeletal pain, pruritus, diarrhea, nausea, asthenia, cough, dyspnea, constipation, decreased appetite, back pain, arthralgia, upper respiratory tract infection, and pyrexia [8,12].

### 4.2. Pembrolizumab

Most common AEs (reported in ≥20% of patients) include fatigue, cough, nausea, pruritus, rash, decreased appetite, constipation, arthralgia, and diarrhea [9,13].

### 4.3. Durvalumab

Most common AEs (≥15% of cases) are fatigue, musculoskeletal pain, constipation, decreased appetite, nausea, peripheral edema, and urinary tract infection [14].

### 4.4. Ipilimumab

Most common AEs (≥5% of cases) are fatigue, diarrhea, pruritus, rash, and colitis [16]. Additional common adverse reactions at the 10 mg/kg dose (≥5%) include nausea, vomiting, headache, weight loss, pyrexia, decreased appetite, and insomnia [16].

### 4.5. Avelumab

Most common AEs (reported in ≥20% of patients) are fatigue, musculoskeletal pain, diarrhea, nausea, infusion-related reaction, rash, decreased appetite, and peripheral edema [17,18].

### 4.6. Atezolizumab

The most common AEs (reported in ≥0% of patients) wae fatigue/asthenia, decreased appetite, nausea, cough, and dyspnea [19].

### 4.7. Tremelilumab

The profile of AEs associated with tremelimumab mainly includes gastrointestinal, skin, and endocrine disorders, recorded as mild and reversible [20].

## 5. Immune Check-Point Inhibitors in Addition to (Chemo)Radiotherapy: Pros and Cons

There are several theoretical advantages in integrating immune check-point inhibitors with standard chemoradiotherapy in HNC. Based on the assumption that ionizing radiation (i) enhances dendritic cell and T cell activation and proliferation and (ii) disrupts tumor architecture, giving RT before or concurrently with checkpoint blockade, anti-PD-1 antibody might benefit from an increase in PD-1 expression on T cells, promoting a superior tumor control [3]. On the other hand, chemoradiotherapy may contribute to a severe immunosuppression status, as well as to changes in tumor microenvironment pH and tissue oxygenation. These circumstances may exacerbate the severe acute and late toxicity—such as swallowing dysfunction, chronic xerostomia, osteroadionecrosis [22,23,24]—and therefore negatively impact on morbidity and ultimately increase the risk of non-cancer mortality.

A hypofractionated RT scheme represents another promising strategy for combining immune check-point inhibitors and radiation therapy [3]. A high dose administered to a small target volume could minimize damage to circulating blood, sparing circulating lymphocytes while supporting anti-PD-1 antibody activity.

Lastly, human papilloma virus (HPV) status may impact the response to therapy, because of its immunologically active tumor microenvironment in HPV-related HNC [25,26]. Some data suggest that immune biomarkers such as the presence of PD-L1+ immune cells can identify subgroups of HPV-positive patients with a strong response to PD-1 or PD-L1 check-point inhibitors [27].

## 6. Clinical Evidence in Head and Neck Cancer

At the time of publication, nivolumab and pembrolizumab are recommended in patients with recurrent/metastatic squamous cell carcinoma of the head and neck, whereas the other molecules are not approved for HNC treatment. However, all these immune check-point inhibitors are currently being tested in different ongoing HNC clinical trials in the curative setting. Details are listed in Table 2 [28,29,30,31,32,33]. To note, two phase III studies, the JAVELIN Head and Neck 100 trial (NCT02952586) and the KEYNOTE-412 (NCT03040999), have been firstly presented at the 2017 ASCO annual meeting and at the 2017 ESMO annual meeting, respectively [34,35]. The Javelin head and neck 100 trial is a phase III randomized, placebo-controlled study planned to evaluate the safety and anti-tumor activity of avelumab in combination with standard chemoradiation versus chemoradiotherapy alone in front-line treatment of patients with locally advanced HNC [34]. In total, 697 patients were randomized to receive three weekly doses of cisplatinum-based chemoradiotherapy with or without avelumab 10 mg/kg on day 1 of the lead-in phase; on days 8, 25, and 39 of the concomitant chemoradiotherapy phase; and every 2 weeks for 12 months during the maintenance phase. Radiation therapy was delivered using the intensity-modulated technique (IMRT) up to 70 Gy (2 Gy per fraction). Primary end point was progression-free survival (PFS). Estimated study completion was June 2020, but the trial was stopped early (in March 2020) due to doubt in primary end-point achievement [35]. Authors reported interim results at the ESMO 2020 [36]. Despite similar tolerability, no improvement in PFS (hazard ratio [HR], 1.21; 95% confidence interval [CI] 0.93–1.57; *p*-value = 0.920); OS (HR 1.31; 95% CI 0.93–1.85; *p*-value = 0.937) was observed with avelumab plus chemoradiotherapy followed by avelumab maintenance versus placebo plus chemoradiotherapy [36].

The KEYNOTE-412 is a phase III, randomized, placebo-controlled, double-blind trial planned to determine efficacy and safety of pembrolizumab plus chemoradiotherapy and as a maintenance therapy versus placebo plus chemoradiotherapy in locally advanced HNC [37]. At present, the study is active, not recruiting. Patients are randomly assigned (1:1) to receive pembrolizumab 200 mg every 3 weeks plus cisplatin-based chemoradiotherapy or placebo plus cisplatin-based chemoradiotherapy [37]. Priming dose of pembrolizumab or placebo is given 1 week before chemoradiotherapy, followed by 2 doses during chemoradiotherapy and an additional 14 doses after chemoradiotherapy, for a total of 17 pembrolizumab or placebo infusions. Radiotherapy consists in an accelerated regimen (56–70 Gy, six fractions per week for 6 weeks) or a standard regimen (56–70 Gy, five fractions per week for 7 weeks). Primary end point is event-free survival. Preliminary data will be presented shortly.

Recently, several trials have been presented at the 2020 ASCO annual meeting. These are the first completed studies testing the addition of an immune check-point inhibitor to curative chemoradiation in head and neck squamous cell carcinoma. Here, we describe their main characteristics and preliminary results.

*Durvalumab.* The CheckRad-CD8 trial (NCT03426657) is a prospective multicenter phase II trial [38]. Patients with squamous cell HNC stage III–IVB received a single cycle of cisplatin (30 mg/mq on day 1–3), docetaxel (75 mg/mq on day 1), durvalumab (1500 mg on day 5), and tremelimumab (75 mg on day 5). In total, 57 patients were enrolled, and 27 patients (47%) had oropharyngeal tumors (52% p16-positive). Preliminary results demonstrated that a single cycle induction treatment with cisplatin, docetaxel, durvalumab, and tremelimumab is feasible and achieved a high pathological complete response rate (47%) in the re-biopsy after the induction treatment [38]. This efficacy seems to be comparable with the rate of clinical complete responses after standard three cycles of docetaxel, cisplatin, and 5-fluorouracil (TPF) induction, ranging from 17% to 43% [39]. Severe toxicity was recorded in 39 patients (68%) and mainly consisted of leucopenia (43%), infections (28%), and immune-related adverse effects (11%). Toxicity profile seems pretty high, but leucopenia and infections may be prevented by the prophylactic use of granulocyte colony-stimulating factor.

*Nivolumab and ipilimumab.* Combined immunotherapy has been tested in HNC management. Authors evaluated nivolumab and ipilimumab in lieu of standard chemotherapy, with concurrent radiotherapy in patients with high-risk locally advanced (stage IVA-IVB, 7th TNM) squamous cell HNC (NCT03162731) [40]. Nivolumab (3 mg/kg every 2 weeks for 17 doses) and Ipilimumab (1 mg/kg every 6 weeks for six doses) were administered starting 2 weeks before radiotherapy. Radiation therapy was prescribed to a dose of 70 Gy (2 Gy per fraction), and the volumetric (VMAT) technique was used. The primary objective was treatment safety. Secondary objectives included 1-year PFS and OS. The combination seemed feasible and has resulted in no loco-regional relapses so far. However, attention should be paid to treatment side effects, potentially linked to the elevated radiosensitizing effect of dual PD-1 and CTLA-4 blockade. Severe acute in-field toxicity occurred in 17/24 (71%) patients (*n* = 9 mucositis, *n* = 6 dysphagia, *n* = 5 dermatitis, *n* = 4 odynophagia, *n* = 1 dysphonia). During the immunotherapy maintenance phase, five patients developed in-field ulcerations at the primary site detected at an average of 3 months post radiotherapy; one of them died of bleeding due to erosion into the carotid artery, with no evidence of active cancer.

## 7. Conclusions

For sure, the role of immune check-point inhibitors in a curative HNC setting remains to be determined. Taken together, these preliminary results support the potential role of these molecules in definitive HNC treatment. Despite these results being preliminary, these immune check-point inhibitors should be considered as an attractive treatment modality that could be added to the arsenal of strategies for the primary therapy of HNC in the near future. At present, there are no data on improved survival outcomes, and toxicity remains a concern when applying a radio–immunotherapy combination. It is possible that the combination of CTLA-4 and PD-1 immune check-point inhibitors would exacerbate synergistic anticancer effects. Additional clinical trials are necessary to investigate new alternative therapeutic strategies that should be well tolerated and that may significantly improve survival outcomes in HNC patients.

## Figures and Tables

**Table 1:** Main characteristics of immune check-point inhibitors in a curative HNC scenario.

Immune Checkpoint Inhibitor	Target	Mode of Action	Toxicity	Clinical Evidence in Curative HNC	Outcomes
Nivolumab	PD-1	It binds to PD-1	fatigue, rash, musculoskeletal pain, pruritus, diarrhea, nausea, asthenia, cough, dyspnea, constipation, decreased appetite, back pain, arthralgia, upper respiratory tract infection, pyrexia	Nivo-Ipi-RT [40]	Safety (primary); PFS, OS
Pembrolizumab	PD-1	It binds to PD-1	fatigue, cough, nausea, pruritus, rash, decreased appetite, constipation, arthralgia, diarrhea	KEYNOTE-412 trial [37]	EFS (primary); OS, safety, and patient-reported outcomes
Avelumab	PD-L1	It binds to PD-L1	fatigue, musculoskeletal pain, diarrhea, nausea, infusion-related reaction, rash, decreased appetite, peripheral edema	JAVELIN Head and Neck 100 trial [36]	PFS (primary); grade ≥3 adverse events
Durvalumab	Anti-PD-L1	It blocks the interaction of PD-L1 with PD-1 and CD80	fatigue, musculoskeletal pain, constipation, decreased appetite, nausea, peripheral edema, urinary tract infection	CheckRad-CD8 trial [38]	Safety (primary); PFS, OS, pathological response
Ipilimumab	CTLA-4	It binds to CTLA-4	fatigue, diarrhea, pruritus, rash, colitis	Nivo-Ipi-RT [40]	Safety (primary); PFS, OS
Atezolizumab	Anti-PD-L1	It binds to the ligand PD-L1 on tumor cells and immune cells	immune-mediated pneumonitis, colitis, hepatitis, endocrinopathies, renal dysfunction, rash, dermatitis	IMvoke010 [33]	EFS e OS (primary); adverse events, patient-reported outcomes
Tremelimumab	CTLA-4	It binds to CTLA-4	gastrointestinal, skin, endocrine disorders		

HNC: head neck cancer; PD-1: programmed cell death protein 1; PD-L1: programmed death-ligand 1; CTLA-4: cytotoxic T lymphocyte antigen 4; EFS: event-free survival; OS: overall survival; PFS: progression-free survival.

**Table 2:** Ongoing trials testing immune check-point inhibitors in addition to definitive chemoradiotherapy in head and neck cancer treatment.

Trial Identifier	Phase	Patient Population	Number Planned	Recruitment Status	Treatment	Primary Outcome
NCT03532737 [28]	II	squamous cell HNC stage III-IVA	50	Recruiting	CRT +/− Pembrolizumab	DLT; RR
NCT03721757 [29]	II	high risk oral cavity cancer	120	Not yet recruiting	Nivolumab before surgery and after adjuvant CRT	DFS; rR
NCT04405154 [30]	II	squamous cell HNC	32	Not yet recruiting	CRT + camrelizumab	Objective RR
NCT03624231 [31]	II	locally advanced HPV-negative HNC	120	Recruiting	Durvalumab-RT +/− tremelimumab	Efficacy, feasibility
NCT03944915 [32]	II	locally advanced HPV-negative HNC	36	Recruiting	Nivoumab + induction chemotherapy (carboplatin-paclitaxel)	Deep RR
NCT03452137 [33]	III	locally advanced squamous HNC	400	Recruiting	Atezolizumab versus placebo as adjuvant therapy after definitive local therapy	EFS, OS

HNC: head and neck cancer; DLT: dose limiting toxicity; RR: response rate; DFS: disease-free survival; rR: recruitment rate; RT: radiotherapy; HPV: human papilloma virus; pCR: pathologic complete response; EFS: event-free survival; OS: overall survival.

## Data Availability

Datasets are available from the corresponding author on reasonable request.
